# Mental health in primary care: an evaluation using the Item Response Theory

**DOI:** 10.11606/S1518-8787.2018052000051

**Published:** 2018-02-07

**Authors:** Hugo André da Rocha, Alaneir de Fátima dos Santos, Ilka Afonso Reis, Marcos Antônio da Cunha Santos, Mariângela Leal Cherchiglia

**Affiliations:** I Universidade Federal de Minas Gerais. Faculdade de Medicina. Programa de Pós-Graduação em Saúde Pública. Belo Horizonte, MG, Brasil; II Universidade Federal de Minas Gerais. Faculdade de Medicina. Departamento de Medicina Preventiva e Social. Belo Horizonte, MG, Brasil; III Universidade Federal de Minas Gerais. Instituto de Ciências Exatas. Departamento de Estatística. Belo Horizonte, MG, Brasil; IV Universidade Federal de Minas Gerais. Faculdade de Medicina. Departamento de Medicina Preventiva e Social. Programa de Pós-Graduação em Saúde Pública. Belo Horizonte, MG, Brasil

**Keywords:** Mental Health Services, organization & administration, Primary Health Care, Patients, classification, Health Services Research, Serviços de Saúde Mental, organização & administração, Atenção Primária à Saúde, Pacientes, classificação, Pesquisa sobre Serviços de Saúde

## Abstract

**OBJECTIVE:**

To determine the items of the Brazilian National Program for Improving Access and Quality of Primary Care that better evaluate the capacity to provide mental health care.

**METHODS:**

This is a cross-sectional study carried out using the Graded Response Model of the Item Response Theory using secondary data from the second cycle of the National Program for Improving Access and Quality of Primary Care, which evaluates 30,523 primary care teams in the period from 2013 to 2014 in Brazil. The internal consistency, correlation between items, and correlation between items and the total score were tested using the Cronbach’s alpha, Spearman’s correlation, and point biserial coefficients, respectively. The assumptions of unidimensionality and local independence of the items were tested. Word clouds were used as one way to present the results.

**RESULTS:**

The items with the greatest ability to discriminate were scheduling of the agenda according to risk stratification, keeping of records of the most serious cases of users in psychological distress, and provision of group care. The items that required a higher level of mental health care in the parameter of location were the provision of any type of group care and the provision of educational and mental health promotion activities. Total Cronbach’s alpha coefficient was 0.87. The items that obtained the highest correlation with total score were the recording of the most serious cases of users in psychological distress and scheduling of the agenda according to risk stratification. The final scores obtained oscillated between -2.07 (minimum) and 1.95 (maximum).

**CONCLUSIONS:**

There are important aspects in the discrimination of the capacity to provide mental health care by primary health care teams: risk stratification for care management, follow-up of the most serious cases, group care, and preventive and health promotion actions.

## RESUMO

**OBJETIVO:**

Determinar os itens do Programa Nacional de Melhoria do Acesso e da Qualidade da Atenção Básica que melhor avaliam a capacidade de oferta de cuidados em saúde mental.

**MÉTODOS:**

Estudo transversal efetuado por meio do Modelo de Resposta Gradual da Teoria da Resposta ao Item, utilizando dados secundários do segundo ciclo do Programa Nacional de Melhoria do Acesso e da Qualidade da Atenção Básica, tendo sido avaliadas 30.523 equipes de atenção básica no período de 2013 a 2014. A consistência interna, a correlação entre os itens e dos itens com o escore total foram testadas usando os coeficientes alfa de Cronbach, correlação de Spearman e ponto bisserial, respectivamente. Foram testados os pressupostos de unidimensionalidade e independência local dos itens. Utilizaram-se como uma das formas de apresentação dos resultados as nuvens de palavras.

**RESULTADOS:**

Programar a agenda de acordo com a estratificação de risco, manter registro dos casos mais graves de usuários em sofrimento psíquico e ofertar algum atendimento em grupo foram os itens com maior capacidade de discriminação. Ofertar alguma modalidade de atendimento em grupo e ofertar ações educativas e de promoção de saúde mental foram os itens que requereram maior nível de oferta de cuidados em saúde mental no parâmetro de locação. O coeficiente alfa de Cronbach total foi 0,87. Realizar o registro dos casos mais graves de usuários em sofrimento psíquico e programar a agenda de acordo com a estratificação de risco foram os itens que obtiveram maior correlação com o escore total. Os escores finais obtidos oscilaram entre -2,07 (mínimo) e 1,95 (máximo).

**CONCLUSÕES:**

São aspectos relevantes na discriminação da capacidade de ofertar cuidados em saúde mental pelas equipes de atenção básica: a estratificação do risco para gestão do cuidado, o acompanhamento dos casos mais graves, o atendimento em grupo e as ações preventivas e de promoção da saúde.

## INTRODUCTION

The introduction of mental health within Primary Health Care (PHC) actions has been a challenge since the primary discussions of the Brazilian Sanitary and Psychiatric Reforms. In Brazil, the Family Health Strategy was chosen as a way to operationalize the PHC^9^.

The World Health Organization (WHO) published the 2001 World Health Report^a^ on the conditions under which the care for persons in psychological distress was provided. The Organization has made indications for the integration of mental health care into primary care.

The WHO launched the Mental Health Gap Action Program (mhGAP)^b^ in 2008. In it, it encourages the implementation of mental health services into primary care in low- and middle-income countries.

The provision of mental health care in PHC ensures the compliance with the principle of integrality, which is the guideline of the PHC and the Brazilian Unified Health System. The PHC plays a key role in the coordination of networks and must ensure to users timely access according to their needs^10^.

The change established by the Psychiatric Reform in the care logic goes from the hospital focus centered on the biomedical model to the care based on the psychosocial care provided to the user in the territory. This implies that the PHC is prepared to ensure the care access of users in psychological distress^3^.

An important factor for the treatment of persons in mental distress by the PHC is the qualification of professionals. These professionals have reported feeling unprepared for such care^13^ ^,^ ^16^ ^,^ ^17^ ^,^ ^21^ ^,^ ^22^.

So that PHC physicians and professionals can prepared, the organization of health systems must be invested on to bring primary and secondary levels closer together, in addition to establishing communication flows and ensuring that primary level professionals receive guidance and supervision at the secondary level^6^ ^,^ ^8^ ^,^ ^22^ ^,^ ^27^. The fragmentation of health systems hinders the access and communication among professionals, causing users not to have continuous care^6^ ^,^ ^24^.

In order to overcome the fragmentation of the care, expressed by the logic of user referral among the network services, the matrix support emerges as a possibility in the Brazilian Unified Health System. The matrix support aims to offer specialized support to primary health care teams (PHCT), allowing the user to be treated by the PHCT to which he or she is linked^4^. Thus, we can avoid the loss of the link of the user with the team and the health unit, receiving treatment close to his or her household.

The National Program for Improving Access and Quality of Primary Care (PMAQ-AB)^c^ was established in 2011 to improve the quality of the PHC and increase the access to it. Mental health care is part of the evaluation of the PHCT, expressed in a sub-dimension of the external evaluation questionnaire. The analysis of the mental health items allows the classification of the PHCT regarding the level of care provided.

This study aimed to verify which items that make up the PMAQ-AB better discriminate the capacity to provide mental health care by the primary health care teams.

## METHODS

This is a cross-sectional study performed using data from the second cycle of the PMAQ-AB, related to 30,523 PHCT. We collected the data from the external evaluation phase of 2013 and 2014. The information from the external evaluation supports the evaluation of the conditions of access and quality of the teams participating in the program.

Not all PHCT enrolled participated in the entire evaluation cycle. Therefore, we excluded the teams that were disqualified (which did not undergo the external evaluation) (n = 713) or classified as unsatisfactory (which did not comply with the contractual commitments) (n = 353).

The analysis of the variables was based on the Item Response Theory (IRT), using the Samejima’s Graded Response Model^26^. The IRT is used to measure non-observable characteristics (latent trait, ability, aptitude, or construct) from observable variables^23^. We defined the ability of a team to provide mental health care as a construct to be measured.

A set of items was selected based on the subdivision proposed by the Ministry of Health (MH) in the external evaluation instrument. The initial analysis of this set of items using the IRT showed that the proposed subdivision is incapable of satisfactorily discriminating the teams in relation to the provision of mental health care.

For the new subdivision, we searched for the term “mental health” throughout the questionnaire of Module II. The variables found were grouped with the others. We selected dichotomous and polytomous variables. All variables were recoded to express the gradation between the worst and the best scenario.

After preliminary analysis of the 25 variables (23 dichotomous and two polytomous variables), six did not fit the model, because they interfered with the assumption of independence or because they were difficult to understand. We chose to remove them from the analyses. They were: treatment of persons in psychological distress by the team; use of some specific strategy by the team to take care of cases: others; use of some specific strategy by the team to take care of cases: no specific strategy is carried out; record of the number of the most serious cases of users in psychological distress by the primary care team; record of users with a need from the use of crack cocaine, alcohol, and other drugs by the primary care team; record by the team of users who chronically use benzodiazepines, antipsychotics, anticonvulsants, antidepressants, mood stabilizers, as well as anxiolytics in general.

Among the 19 remaining variables, 17 were dichotomous and two were characterized in three levels. We recoded the two selected polytomous variables in order to present three graded scenarios. The first variable referred to the way appointments are scheduled for persons in psychological distress. The best scenario was the answer: “appointments are scheduled on any day of the week, at any time.” In the intermediate scenario, we grouped the answers: “any day of the week, at specific times”, “specific fixed days, at any time”, “specific fixed days, at specific times”, “other”; and in the worst case scenario, we placed the answer “no appointments are scheduled”.

The second polytomous variable was related to waiting time for the first care service of persons in psychological distress. We considered the answer “treated on the same day” as the best scenario. In the intermediate scenario, we grouped the answers that ranged from one day to 270 days of waiting; and in the worst case, we placed the answer “no service.”

We used the statistical software R^d^ for the adjustments to the Samejima’s Graded Response Model (GRM), with the aid of the ltm package^25^. The GRM enables the estimation of the parameters of discrimination (parameter a) and location (parameter b) for each answer category of the item^26^. We used the discrimination parameter to identify the items of the questionnaire with greater power of discrimination for the teams in relation to the level of the construct under study, that is, to distinguish the teams with greater capacity to “provide mental health care”.

In the model used, the location parameter reached by a team is equivalent to the latent trait level in which the probability of choosing the category or a higher one is 50%. The values of this parameter are usually located between -3 and 3, the same scale of the scores obtained by the teams^1^.

The Cronbach’s alpha, Spearman’s correlation, and point biserial coefficients were respectively used to analyze internal consistency, correlation between items, and correlation of items with total score.

The Item Characteristic Curves (ICC) were used to verify the probability of choice for each of the categories of the items as a function of the capacity to “provide mental health care”.

The IRT has as assumptions the unidimensionality (the items must measure a single latent trait) and the local independence of the items (given a score, the answers to the items cannot show dependence among themselves). We used the Principal Component Analysis to evaluate unidimensionality^1^ ^,^ ^12^.

After adjusting the GRM to the data, we could estimate the parameters of the items and the scores related to the “capacity to provide mental health care” reached by each team. We carried out the descriptive analysis of the scores, using frequency distribution and the measures of central tendency and dispersion. The scores were kept on the usual IRT scale (-3 to 3) and divided into four groups: -3 to -1.5 (far below average), -1.5 to 0 (below average), 0 to 1.5 (above average), and 1.5 to 3 (far above average).

We used word clouds for the visualization of the frequency of the answers given to the items by the teams, one for each group. We assigned microtexts to the answer alternatives of each item. The microtexts were used to illustrate the answers that mostly started with the letters “Y” (yes) and “N” (no). The font size of the terms in the word cloud was directly related to how often we found the term. The terms typed with the same colors had a close answer frequency.

This study complied with the regulatory guidelines and standards of research involving humans established in Resolution 466 of December 12, 2012. The research was approved by the Research Ethics Committee of Universidade Federal de Minas Gerais (Record 28804 on May 30, 2012).

## RESULTS

The variables related to the provision of group care, provision of educational and health promotion actions, performance of actions for persons with a need from the use of drugs, and the record of users with a need from the use of drugs obtained a higher proportion of answers in the worst-case scenario (Table 1).

**Table 1:** Distribution of the proportion of each answer category. Brazil, 2013–2014.

Item	Proportion of answers (%)		

	1^a^	2	3
1 Protocols with therapeutic guidelines	52.9	47.1	n.a.
2 Programming of the provision of appointments	44.8	55.2	n.a.
3 Protocols for risk stratification	50.4	49.6	n.a.
4 Programming of the schedule according to stratified risk	53.9	46.1	n.a.
5 Maintenance of the record of users referred	53.7	46.3	n.a.
6 Performance of an active search	51.2	48.8	n.a.
7 Scheduling of appointments^b^	11.7	23.1	65.14
8 Waiting time for the first care service^c^	11.7	25.8	62.42
9 Provision of appointments with more time	52.7	47.3	n.a.
10 Use of record of life history	54.8	45.2	n.a.
11 Provision of a group care service	72.4	27.6	n.a.
12 Care with matrix support professionals	37.9	62.1	n.a.
13 Preparation for the care service	60.3	39.7	n.a.
14 Record of the most serious cases of users in psychological distress	46.9	53.1	n.a.
15 Record of users with a need from drug use	63.1	36.9	n.a.
16 Performance of actions for persons with a need from drug use by the team	63.3	36.7	n.a.
17 Recording record of users who chronically use psychotropic substances by the team	44.8	55.2	n.a.
18 Performance of actions for persons who chronically use psychotropic substances by the team	51.1	48.9	n.a.
19 Provision of educational and health promotion actions by the team	67.5	32.5	n.a.

^a^ Dichotomous variables: 1 = No; 2 = Yes; n.a. = not applicable.

^b^ Item 7 - Scheduling of appointments: 1 = no appointments are scheduled; 2 = they are scheduled “any day of the week, at specific times”, “specific fixed days, at any time”, “specific fixed days, at specific times”, “other”; 3 = they are scheduled on any day of the week, at any time.

^c^ Item 8 - Waiting time for the first care service: 1 = no service; 2 = it is scheduled from 1 to 270 waiting days; 3 = the service is scheduled for the same day.

On the other hand, the scheduling of appointments, the waiting time for the first care service, and the service with matrix support professionals obtained a higher proportion of answers in the best-case scenario.

Considering all the items, we obtained Cronbach’s alpha coefficient of 0.87, which did not improve with the removal of any item. The correlation of the items with the final score ranged from 0.35 (item 8) to 0.63 (item 14) (Table 2).

**Table 2:** Estimates of the parameters of discrimination and location, internal consistency, and correlation between items and total score. Brazil, 2013–2014.

Item	Parameters (standard error)			Total internal consistency (0.87)	Correlation with total score

	a^a^	b1^b^	b2^c^		
1 Protocols with therapeutic guidelines	1.52 (0.02)	0.12 (0.01)	n.a.	0.86	0.57
2 Programming of the provision of appointments	1.47 (0.02)	-0.18 (0.01)	n.a.	0.86	0.56
3 Protocols for risk stratification	1.56 (0.02)	0.03 (0.01)	n.a.	0.86	0.58
4 Programming of the schedule according to stratified risk	1.79 (0.03)	0.15 (0.01)	n.a.	0.86	0.62
5 Maintenance of the record of users referred	1.47 (0.02)	0.15 (0.01)	n.a.	0.86	0.56
6 Performance of an active search	1.29 (0.02)	0.06 (0.01)	n.a.	0.86	0.52
7 Scheduling of appointments	1.12 (0.02)	-2.13 (0.03)	-0.62 (0.02)	0.86	0.41
8 Waiting time for the first care service	0.91 (0.02)	-2.48 (0.04)	-0.56 (0.02)	0.86	0.35
9 Provision of appointments with more time	1.44 (0.02)	0.12 (0.01)	n.a.	0.86	0.55
10 Use of record of life history	1.59 (0.03)	0.19 (0.01)	n.a.	0.86	0.58
11 Provision of a group care service	1.72 (0.03)	0.84 (0.01)	n.a.	0.86	0.55
12 Care with matrix support professionals	1.24 (0.02)	-0.50 (0.01)	n.a.	0.86	0.48
13 Preparation for the care service	1.15 (0.02)	0.47 (0.01)	n.a.	0.86	0.47
14 Record of the most serious cases of users in psychological distress	1.79 (0.03)	-0.09 (0.01)	n.a.	0.86	0.63
15 Record of users with need from drug use	1.61 (0.03)	0.49 (0.01)	n.a.	0.86	0.57
16 Performance of actions for persons with a need from drug use by the team	1.45 (0.02)	0.53 (0.01)	n.a.	0.86	0.54
17 Recording record of users who chronically use psychotropic substances by the team	1.39 (0.02)	-0.19 (0.01)	n.a.	0.86	0.54
18 Performance of actions for persons who chronically use psychotropic substances by the team	1.69 (0.03)	0.06 (0.01)	n.a.	0.86	0.61
19 Provision of educational and health promotion actions by the team	1.53 (0.03)	0.68 (0.01)	n.a.	0.86	0.54

n.a.: not applicable

^a^ Discrimination.

^b^ Location (answer 2).

^c^ Location (answer 3).

**Figure 1:**
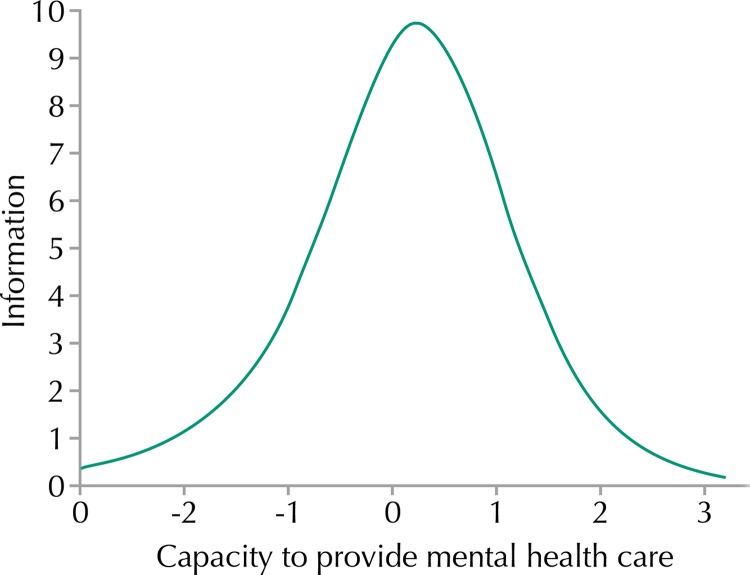
Total Information Curve. Brazil, 2013–2014.

The items with greater power of discrimination were scheduling of the agenda according to risk stratification, keeping of records of the most serious cases of users in psychological distress, and provision of group care, with the values of 1.79, 1.79, and 1.72, respectively. The items that presented less power of discrimination were those categorized into three levels: scheduling of appointments (1.12) and waiting time for the first service care (0.91). The Total Information Curve is shown in Figure 1.

Regarding the parameter of item location, we obtained the highest values for the provision of group care (0.84) and provision of educational and mental health promotion actions (0.68).

The principal component analysis, used to evaluate the assumption of unidimensionality of the items, showed a marked reduction in the percentage of variability between the first (43.3%) and second (9.9%) components, which indicates that the assumption of unidimensionality was met.

The calculation of the scores of the level of provision of mental health care presented values between -2.07 (minimum) and 1.95 (maximum). The average was 0.006 and the median was 0.032 (Table 3).

**Table 3:** Distribution according to the score ranges of the provision of mental health care. Brazil, 2013–2014.

Scores – level	Absolute frequency	Relative frequency

	n	%
(-3.0 to -1.5) - Far below average	1,777	6.0
(-1.5 to 0.0) - Below average	12,386	42.0
(0.0 to 1.5) - Above average	14,016	47.6
(1.5 to 3.0) - Far above average	1,278	4.3

Total	29,457	100

Yprotocol: presence of protocols to receive the spontaneous demand; Nprotocol: no presence of protocols to receive the spontaneous demand; Yprog_appoint: programming of the provision of appointments; Nprog_appoint: no programming of the provision of appointments; Yprot_risk_stratif: use of protocols for risk stratification; Nprot_risk_stratif: no use of protocols for risk stratification; Yprog_schedule: programming of the schedule according to the classified risk; Nprog_schedule: no programming of the schedule according to the classified risk; Ymaintenance_record: maintenance of the record of higher risk users referred; Nmaintenance_record: no maintenance of the record of higher risk users referred; Yactivesearch: performance of an active search; Nactivesearch: no performance of an active search; Sched_appointsameday: scheduling of the appointment on the same day; Sched_appointspecific: scheduling of the appointment on specific conditions; Nscheduling: no scheduling of appointments; Careservicesameday: care service happens on the same day; +1daywaiting: care service happens in more than one waiting day; Doesnotcareservice: care service does not happen; Yappointwith+time: use of appointments with more time; Nappointwith+time: no use of appointments with more time; Yreclifehistory: use of record of life history; Nreclifehistory: no use of record of life history; Yprovgroup: provision of group service; Nprovgroup: no provision of group service; Ymatrixsupport: performance of the care service with professional matrix support; Nmatrixsupport: no performance of the care service with professional matrix support; Ypreparation: preparation for the care service; Npreparation: no preparation for the care service; Yrec+seriouscases: presence of the record of the most serious cases; Nrec+seriouscases: no presence of the record of the most serious cases; Yrec_usersusedrugs: presence of the record of the users with a need from the use of drugs; Nrec_usersusedrugs: no presence of the record of the users with a need from the use of drugs; Yactionsusersdrugs: performance of actions for persons with a need from the use of drugs; Nactionsusersdrugs: no performance of actions for persons with a need from the use of drugs; Yrecchronicpsychdrugs: presence of the record of users who chronically use psychotropic drugs; Nrecchronicpsychdrugs: no presence of the record of users who chronically use psychotropic drugs; Yactionschronicpsychdrugs: performance of actions for persons who chronically use psychotropic drugs; Nactionschronicpsychdrugs: no performance of actions for persons who chronically use psychotropic drugs; YhealthpromMH: provision of educational and health promotion actions; NhealthpromMH: no provision of educational and health promotion actions

**Figure 2:**
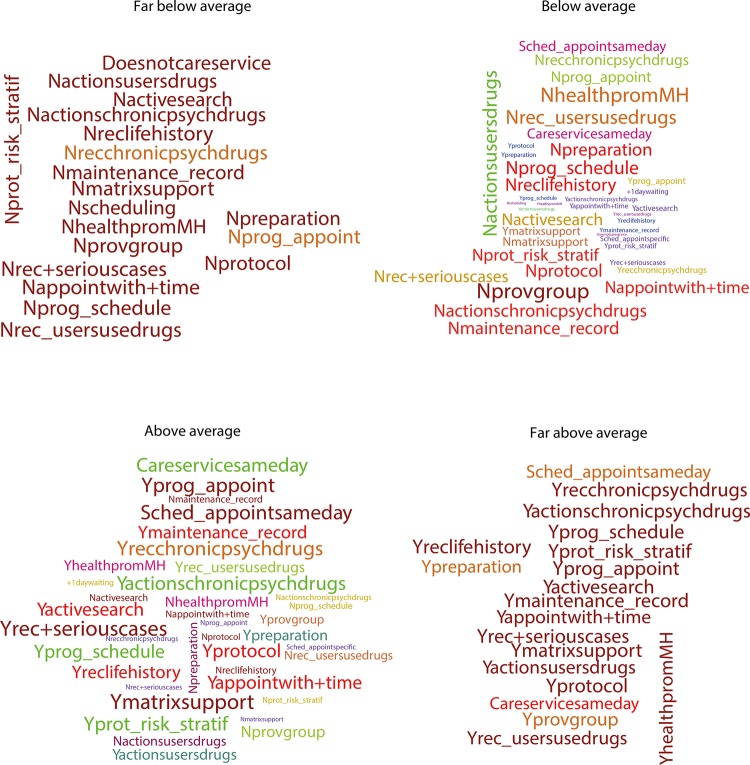
Characteristics of the teams by score range. Brazil, 2013–2014.

Figure 2 shows word cloud diagrams representing the frequency distribution of the answer categories of the four groups of teams with different levels of provision of mental health care.

At the levels of provision of care of far below average and far above average, the frequencies were uniform, evidenced by the fact that the words had similar sizes.

At the level of far above average, answers categorized as best-case scenario were predominant (microtexts starting with “Y”). On the other hand, at the level of far below average, the answers in the worst-case category had many records (microtexts starting with “N”).

At levels of below average and above average, we observed a great heterogeneity of the frequency of the answers. Few terms were written with greater intensity, with a pronounced concentration of answers of the best-case scenario in the group above average and answers of the worst-case scenario in the group below average.

When comparing the results presented in the word clouds with the parameters obtained in the items, those items with the highest power of discrimination were differently present at the levels far below average and far above average. The item with the highest power of discrimination, programing of schedules according to stratified risk, was found positively at the level of far above average (Yprog_shedule) and negatively at the level of far below average (Nprog_schedule). The same thing happened with the record of the most serious cases of users in psychological distress, which appeared positively at the level of far above average (Yrec+seriouscases) and negatively at the level of far below average (Nrec+seriouscases).

## DISCUSSION

The selected items presented good capacity to differentiate the PHCT participating in the PMAQ-AB in relation to the provision of mental health care. The most important factors in discriminating the level of provision of mental health care were risk stratification for management care, follow-up of the most serious cases, group care, and prevention and mental health promotion actions.

The selection of variables that went beyond the sub-dimension proposed by the MH in the questionnaire favored the elaboration of the scale, since several questions related to the mental health subject were present in other sub-dimensions of the instrument. As a result, there was an increase in the capacity to evaluate the PHCT in relation to the provision of mental health care.

The elaboration of a scale to measure the provision of mental health care from the assumptions of the IRT presents an important advantage in relation to the Classical Item Analysis Theory. The measurement of the latent trait is not influenced by the level of aptitude of the respondent group, nor by characteristics of the items, which is a property denominated invariance of the parameters^2^ ^,^ ^12^ ^,^ ^23^. Thus, we can infer that the scale obtained by this study is appropriate to evaluate the PHCT. In addition, it can be used to evaluate the behavior of the team over time in the context of continuity of the PMAQ-AB.

When evaluating the items that presented the highest individual correlation with total score – programming of the schedule according to stratified risk, record of the most serious cases of users in psychological distress, and provision of actions for persons who chronically use psychotropic drugs –, we verified that the first two have a closer relationship with the management of health services than with direct user care. This fact is in line with the evaluation dynamics of the PMAQ-AB, which aims to induce teams to continuously improve the quality of service provision and access, with a strong focus on processes^e^.

The record of users in psychological distress is an important indicator for planning both follow-up actions and promotion actions in mental health, helping decision making. Jucá et al.^18^ indicate the absence of records as a reflection of the lack of preparation of the PHCT to handle these users.

In England, general practitioners are evaluated by a performance payment program that monitors them in relation to the care and recording of clinical data of persons in mental distress. In addition to caring for mental disorders, physicians should be aware of factors such as blood pressure, glucose, and cholesterol measurement. Care is not restricted to the symptoms of psychological distress and must be integral^20^.

The provision of group service was the item that presented the highest value for the parameter of location. This shows that the teams that responded positively to this question are more likely to have a high capacity to provide mental health care. Group practices are mechanisms that can favor the follow-up of persons in mental distress in PHC, based on the establishment of bonds and active participation of users^7^. The groups present great potential to be used as a therapeutic tool and as a health promotion strategy. The alignment of the provision of group practices with the promotion of mental health in PHC can favor the establishment and maintenance of longitudinality in the treatment of patients in psychological distress, with a closer relationship between health professionals and users and the achievement of better results^28^.

A Brazilian study indicates that, when guided by the psychosocial model, the groups within the scope of the PHC are effective as actions consistent with the precepts of the Brazilian Psychiatric Reform, promoting the autonomy and the singularity of users^21^.

Traditionally, group practices in PHC are a proposal for intervention for specific audiences, such as diabetic, hypertensive, and obese persons. Despite this, many teams have difficulties in working with this approach, pointing to challenges such as lack of personal ability, lack of resources and inputs, and excessive demand. We can also mention difficulties regarding the operationalization of groups, and many end up becoming “collective appointments”, based on prescriptions and indication of conduct^5^.

The care with professionals from some type of matrix support was one of the items that obtained the highest rate of positive answer, which suggests that the strategy of providing specialized support to the PHCT is adopted on a large scale. The matrix support was designed to act comprehensively, from the sharing and co-responsibility for the care of the user to actions of permanent education provided to the PHCT^4^ ^,^ ^15^.

Studies have indicated the need for supervision and training of PHCT for the adequate care of patients in psychological distress^16^ ^,^ ^17^ ^,^ ^22^ ^,^ ^27^. The matrix support would be able to increase the resolutiveness and the effectiveness of the actions, acting together with the PHCT in their territory, subsidizing group activities, health promotion actions, and training of PHC professionals^7^ ^,^ ^11^.

Aligned with the assumptions of the Brazilian Psychiatric Reform, the matrix support inserts itself as a possibility because it breaks with the logic of fragmented care. Its goal is to align all professionals involved in the care, even if they are from different services^4^ ^,^ ^15^.

A potential side effect of mental health insertion in PHC coupled with the lack of supervision may be the high prescription of psychotropic drugs, especially in patients with common mental disorders^14^ ^,^ ^19^ ^,^ ^21^ ^,^ ^29^.

One limitation of this study is the formulation of the closed questions in the data collection questionnaire. This hinders the better qualification of the data, since it restricts the answer option to yes or no.

The provision of mental health care, although understood as part of the scope of the PHC actions and also present in the evaluation program, is not well developed. In order to strengthen the provision of mental health actions by primary health care teams, managerial practices need to be increased, such as risk stratification for care management and record of follow-up of the most serious cases. Care resources should also be developed, such as group care and actions for the prevention and promotion of mental health.

## Footnotes

a World Health Organization. The world health report 2001- Mental Health: new understanding, new hope. Geneva: WHO; 2001 [cited 2017 Oct 20]. Available from: http://www.who.int/whr/2001/en/

b World Health Organization. mhGAP Mental Health Gap Action Programme: scaling up care for mental, neurological, and substance use disorders. Geneva: WHO; 2008 [cited 2017 Oct 20]. Available from: http://www.who.int/mental_health/evidence/mhGAP/en/

c Ministério da Saúde (BR). Portaria n° 1.654, de 19 de julho de 2011. Institui, no âmbito do Sistema Único de Saúde, o Programa Nacional de Melhoria do Acesso e da Qualidade da Atenção Básica (PMAQ-AB) e o Incentivo Financeiro do PMAQ-AB, denominado Componente de Qualidade do Piso de Atenção Básica Variável – PAB Variável. *Diario Oficial da Uniao*. 20 jul 2011; Seção 1:79.

d R Core Team. R: a language and environment for statistical computing. Version 3.3.1. Vienna: R Foundation for Statistical Computing; 2016.

e Ministério da Saúde (BR), Secretaria de Atenção à Saúde, Departamento de Atenção Básica. Nota metodológica da certificação das equipes de atenção básica participantes do Programa Nacional de Melhoria do Acesso e da Qualidade da Atenção Básica 2013-2014. Brasília (DF); 2015. Available from: http://189.28.128.100/dab/docs/portaldab/documentos/nota_Metodologica_certificacao_eab.pdf
